# Complexation Strategies for Enhancing Water-Soluble Protein Stability and Functionality: A Comprehensive Review

**DOI:** 10.3390/foods14132359

**Published:** 2025-07-02

**Authors:** Qing Guo, Xu Qi, Yuying Hu

**Affiliations:** 1 State Key Laboratory of Food Nutrition and Safety, Tianjin University of Science & Technology, Tianjin 300457, China; qx010312@163.com; 2 School of Food Science and Technology, Tianjin University of Science & Technology, Tianjin 300457, China; 3 School of Life and Health Sciences, Hubei University of Technology, Wuhan 430068, China; huyuying@hbut.edu.cn

**Keywords:** water-soluble protein, complexation, stability, functionality

## Abstract

Water-soluble proteins, as ideal ingredients in food systems, have attracted much attention. However, their applications in the food industry are still limited by inherent shortcomings in stability and functionalities. Complexation with ligands is an effective strategy to overcome these limitations. In the present review, we summarize (1) the interaction mechanisms between water-soluble proteins and ligands; (2) the enhancement of the stability and functionalities of water-soluble proteins; (3) the influences of the nature of proteins and ligands, along with complexing conditions, on their interactions and enhancement of the stability and functionalities of water-soluble proteins; and (4) the applications of water-soluble protein-based complexes. Promising future research trends in the field of water-soluble protein-based complexes are also discussed. This review aims to offer theoretical guidance and technical support for the precise enhancement of protein stability and functionalities, thereby promoting their broader application in the food industry.

## 1. Introduction

Water-soluble proteins are widely distributed in natural sources [1,2,3,4]. Their biocompatibility, nutritional value, and functionality make them important ingredients in the food industry [5]. However, individual water-soluble proteins often possess limited functionalities and exhibit poor stability, particularly at pHs near their isoelectric points or under extreme environmental conditions. These limitations hinder their broader application in the food industry.

The functionalities of water-soluble proteins include physicochemical functionalities and bioactive properties. The physicochemical functionalities refer to the physical, chemical, or processing-related properties of water-soluble proteins, such as emulsifying properties. The bioactive properties refer to the biological effects at the physiological or health level, such as antioxidant activity. Complexation with ligands (such as polysaccharides, polyphenols, proteins, and small-molecule surfactants) to form water-soluble protein–ligand complexes is one of the important strategies for enhancing the stability and functionalities of water-soluble proteins to overcome the aforementioned limitations [6,7,8,9,10]. Interactions between water-soluble proteins and ligands are the basis of complex formation. Different complexing conditions, including the nature of water-soluble proteins, ligands, and complexing conditions, are key factors affecting the enhancement of the stability and functionalities of water-soluble proteins. Publications on the design and fabrication of water-soluble protein-based complexes to enhance stability and functionalities, based on interaction mechanisms between proteins and ligands and the optimization of relevant factors, have increased in the last decade. Conducting a systematic review of these studies to gain deeper insights into the interaction mechanisms between water-soluble proteins and ligands, as well as the influence of complexing conditions on the interactions and enhancement of the stability and functionalities of water-soluble proteins, is essential for the future development of water-soluble protein-based complexes. However, there have been few comprehensive overviews in this field so far. Hence, this review comprehensively summarizes the interaction mechanisms between water-soluble proteins and ligands; the enhancement of the stability and functionalities of water-soluble proteins; the influences of the nature of proteins and ligands, along with complexing conditions, on their interactions and enhancement of the stability and functionalities of water-soluble proteins; and the applications of water-soluble protein-based complexes. This review provides theoretical guidance and technical support for precisely and effectively enhancing the stability and functionalities of water-soluble proteins, thereby promoting their broader application in the food industry.

## 2. Water-Soluble Protein-Based Binary Complexes

Water-soluble proteins can complex with various kinds of ligands through non-covalent interactions (such as hydrogen bonding, electrostatic interactions, and hydrophobic interactions) and covalent interactions (such as Maillard reactions) to form water-soluble protein-ligand complexes [11]. Due to the diverse nature of ligands, they interact with water-soluble proteins in different ways, resulting in varying effects on the enhancements in stability and functionalities of water-soluble proteins. Therefore, in this section, interactions between water-soluble proteins and various ligands, and the effects of ligand incorporation on the enhancements in stability and functionalities of water-soluble proteins are summarized.

### 2.1. Water-Soluble Protein–Polysaccharide Complexes

Water-soluble proteins are prone to aggregation at pHs near their isoelectric points and under harsh environmental conditions (e.g., high temperature or ionic strength), resulting in reduced stability [12]. Moreover, the functionalities of water-soluble proteins are limited by their inherent properties. For instance, the emulsifying properties of water-soluble proteins are limited by their highly hydrophilic nature. To enhance the stability and functionalities of water-soluble proteins, polysaccharides are frequently used to complex with them through non-covalent or covalent interactions [13]. In this section, the interactions between water-soluble proteins and polysaccharides are elucidated in detail, along with the effects of polysaccharides on enhancing the stability and functionalities of water-soluble proteins.

**Figure 1:**
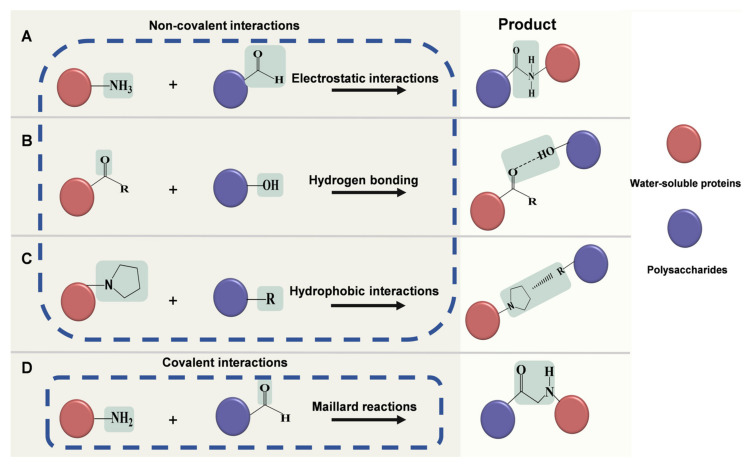
Schematic illustrations of the mechanisms of non-covalent/covalent interactions between water-soluble proteins and polysaccharides. (**A**) Electrostatic interactions. (**B**) Hydrogen bonding. (**C**) Hydrophobic interactions. (**D**) Maillard reactions. “-R” in (**B**,**C**) denotes the other functional groups of water-soluble proteins and the hydrophobic groups of polysaccharides, respectively.

Non-covalent interactions between water-soluble proteins and polysaccharides mainly include electrostatic interactions, hydrogen bonding, and hydrophobic interactions [14]. Covalent interactions between water-soluble proteins and polysaccharides are generally referred to as the initial stage of Maillard reactions [15]. The mechanisms of these interactions are summarized in Box 1 and illustrated in Figure 1. In many cases, the formation of complexes is driven by multiple interactions. For instance, the pea protein isolate–high methoxyl pectin complex is formed through electrostatic interaction and hydrogen bonding [16]. Similarly, whey protein–modified starch complexes are formed through electrostatic and hydrophobic interactions [17]. The predominance of these interactions mainly depends on the complexing conditions and their strength.

Box 1 **The mechanisms of non-covalent/covalent interactions between water-soluble proteins and polysaccharides.**

- Electrostatic interactions occur between the amidogen of water-soluble proteins and the carboxyl of polysaccharides (Figure 1A) [18].
- Hydrogen bonding occurs between the carbonyl oxygen of the amino acid residues of water-soluble proteins and the hydroxyl groups of polysaccharides (Figure 1B) [19].
- Hydrophobic interactions occur between the hydrophobic groups of water-soluble proteins and polysaccharides (Figure 1C).
- Maillard reactions occur between the amino groups of water-soluble proteins and the carbonyl groups of reducing polysaccharides (Figure 1D) [15].

**Figure 2:**
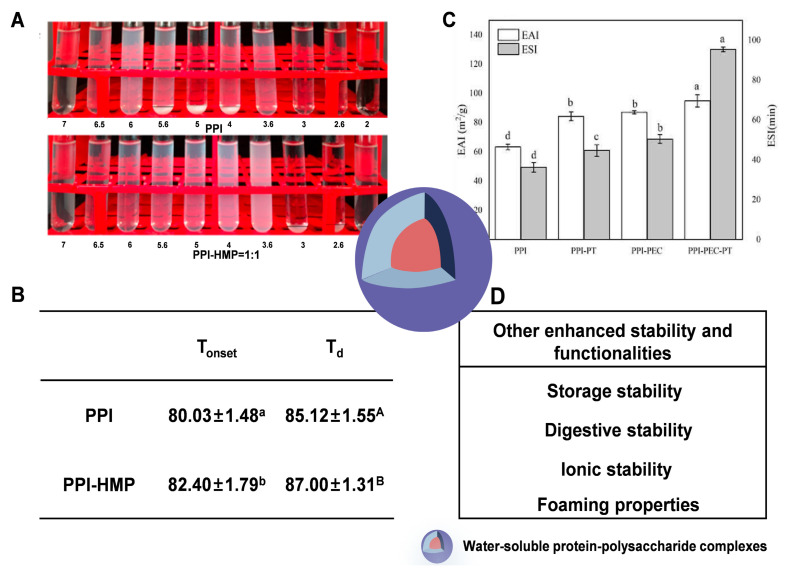
Characterization of the key indicators reflecting the stability and functionalities of water-soluble proteins, in both isolated and complexed forms. (**A**) The visual appearance of water-soluble proteins in both isolated and complexed forms under various pH conditions [16]. (**B**) The thermal denaturation temperatures of water-soluble proteins in both isolated and complexed forms (Different letter symbol indicates that means differ at *p* < 0.05) [16]. (**C**) The emulsifying activity index and emulsifying stability index of water-soluble proteins in both isolated and complexed forms [26]. (**D**) Other enhanced stability and functionalities, where PPI refers to pea protein isolate, HMP refers to high methoxyl pectin, T_onset_ refers to the onset denaturation temperature, T_d_ refers to the peak denaturation temperature, PT refers to pterostilbene, PEC refers to pectin, EAI refers to the emulsification activity index, and ESI refers to the emulsification stability index. Reprinted with permission from the authors.

When water-soluble proteins complex with polysaccharides through non-covalent or covalent interactions, their aggregation at pHs near their isoelectric points is prevented, resulting in enhanced aggregation stability (Figure 2A) [16]. This is because the incorporation of polysaccharides increases steric hindrance and electrostatic repulsion, thereby preventing the aggregation of water-soluble proteins [20]. Polysaccharides commonly used to complex with water-soluble proteins to enhance the aggregation stability of water-soluble proteins at pHs near their isoelectric points include pectin [21], xanthan gum [22], and carrageenan [23]. Moreover, the storage, digestive, pH, and ionic stabilities of water-soluble proteins can also be enhanced after complexation with polysaccharides due to increased steric hindrance or electrostatic repulsion.

The thermal stability of water-soluble proteins is also enhanced after complexation with polysaccharides through non-covalent or covalent interactions. Compared to the thermal denaturation temperature of pea protein isolate (85.12 °C), the pea protein isolates in pea protein isolate-high methoxyl pectin complexes formed through non-covalent interaction has a higher thermal denaturation temperature (87.00 °C), indicating that the thermal stability of pea protein isolate is enhanced by complexation with high methoxyl pectin (Figure 2B) [16]. Similarly, the lactoferrin in the lactoferrin–dextran conjugates also showed a higher thermal denaturation temperature [24]. For proteins in the protein–polysaccharide non-covalent complexes, the enhancement of thermal stability is attributed to the hydrogen bonds formed by them with polysaccharides. The formation of hydrogen bonds is an exothermic process; thus, it takes more energy to induce the protein denaturation [6]. As for protein in protein–polysaccharide covalent complexes, a molecular crowding effect, induced by heating, is proposed to explain the avoidance of the thermal denaturation of proteins under thermal conditions [13].

Generally, it is hard for most water-soluble proteins to effectively reduce the interfacial tension between water and oil due to their high hydrophilicity, resulting in them being hard to adsorb onto oil droplets. Complexation with substituted polysaccharides through non-covalent or covalent interactions can induce conformational changes in water-soluble proteins, leading to the exposure of additional hydrophobic groups or the grafting of new hydrophobic moieties. This enhances the emulsifying activity of water-soluble proteins. Additionally, the presence of abundant charges induced by the incorporation of substituted polysaccharides can strengthen the electrostatic repulsion between emulsion droplets, which inhibits their aggregation and consequently improves the emulsifying stability of water-soluble proteins [25]. The enhanced emulsifying activity and stability contribute to the enhanced emulsifying properties of water-soluble proteins. For instance, the emulsifying activity and stability of pea protein isolate are enhanced after complexation with pectin (Figure 2C), indicating that the emulsifying properties of pea protein isolate can be enhanced by complexation with pectin [26].

The main types of interactions and enhancements in stability and functionalities of water-soluble proteins after complexation with other polysaccharides are summarized in Table 1.

### 2.2. Water-Soluble Protein–Protein Complexes

The fabrication of water-soluble protein-protein complexes also seems to be an ideal way to enhance the stability and functionalities of water-soluble proteins. At present, water-soluble protein–protein complexes in the food field are primarily prepared through non-covalent interactions, including electrostatic interactions, hydrophobic interactions, and hydrogen bonding.

**Figure 3:**
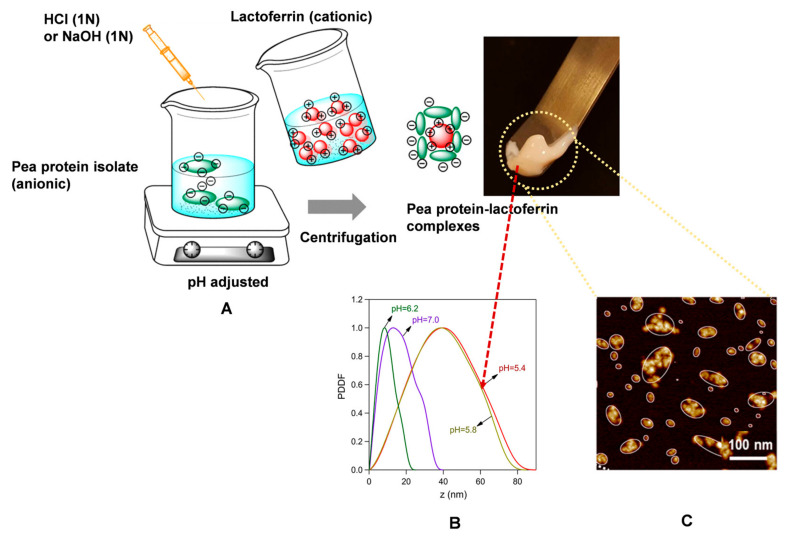
Schematic illustrations of the preparation and characterization of pea protein isolate–lactoferrin complexes [39]. (**A**) The steps for the preparation of pea protein isolate–lactoferrin complexes, showing the visual aspect of the viscous phase. (**B**) The corresponding pair–distance distribution functions (PDDFs) evaluated based on indirect Fourier transformation, showing that the largest complex formed at pH = 5.4. (**C**) An atomic force microscopy topography image of complexes at pH 5.4, with software fits ellipses to each complex. Reprinted with permission from the authors.

The electrostatic interactions between water-soluble proteins and other proteins generally occur between polar amino acids with the opposite charges of the two proteins. Lactoferrin is a ligand commonly used to complex with water-soluble proteins through electrostatic interactions. This is because lactoferrin has a high isoelectric point (8.6), allowing it to carry positive charges at neutral or mildly acidic pH values, thereby enabling electrostatic interactions with negatively charged water-soluble proteins whose isoelectric points are much lower. For instance, pea protein isolate could form complexes with lactoferrin through electrostatic interactions (Figure 3A), and paste-like complexes with the largest size were observed at pH 5.4 (Figure 3B). The atomic force microscopy topography image of complexes at pH 5.4 is shown in Figure 3C [39]. Based on this result, we speculate that complexation with lactoferrin may enhance the gelation properties of pea protein isolate at pH 5.4. However, this assumption still requires further experimental verification.

Generally, it is difficult for water-soluble proteins and ligands carrying the same type of charge to form complexes at room temperature due to electrostatic repulsion. However, as the temperature rises, both protein molecules gradually unfold, exposing hydrophobic groups and enhancing hydrophobic interactions between them. Water-soluble protein–protein complexes are formed when hydrophobic interactions are stronger than repulsive forces between two proteins. Casein is one of the most commonly used ligand proteins in such systems. Its micellar structure resembles that of low-molecular-weight heat shock proteins (chaperone proteins). Complexation with casein can prevent protein aggregation through its chaperone-like activity, thereby enhancing the thermal stability of water-soluble proteins [40]. Specifically, water-soluble protein molecules first unfold and subsequently aggregate via hydrophobic interactions during thermal treatments. Casein can competitively complex with the unfolded protein molecules, thus preventing aggregation. Therefore, casein is regarded as an ideal ligand for enhancing the thermal stability of water-soluble proteins.

In addition to water-soluble proteins, prolamins can also serve as ligands to form complexes with primary water-soluble proteins. For instance, zein has been successfully used as a ligand to form complexes with whey protein isolate via a pH-driven method, and hydrogen bonding has been identified as the main driving force for complex formation. The thermal stability of whey protein isolate has been enhanced by complexation with zein. The authors believed that the enhanced thermal stability of whey protein isolate might be attributed to the formation of a more compact structure, induced by its interaction with zein, which was less susceptible to disintegration during thermal treatment [41]. In addition to thermal stability, the hydrophobicity of water-soluble proteins can be enhanced by complexation with prolamins, further enhancing their emulsifying properties. At present, the design and fabrication of water-soluble protein–protein complexes are still a relatively new topic. Understanding the mechanism of complex formation and the effect of ligand proteins on enhancements in the stability and functionalities of water-soluble proteins will open enormous opportunities for their use in foods.

### 2.3. Water-Soluble Protein–Polyphenol Complexes

**Figure 4:**
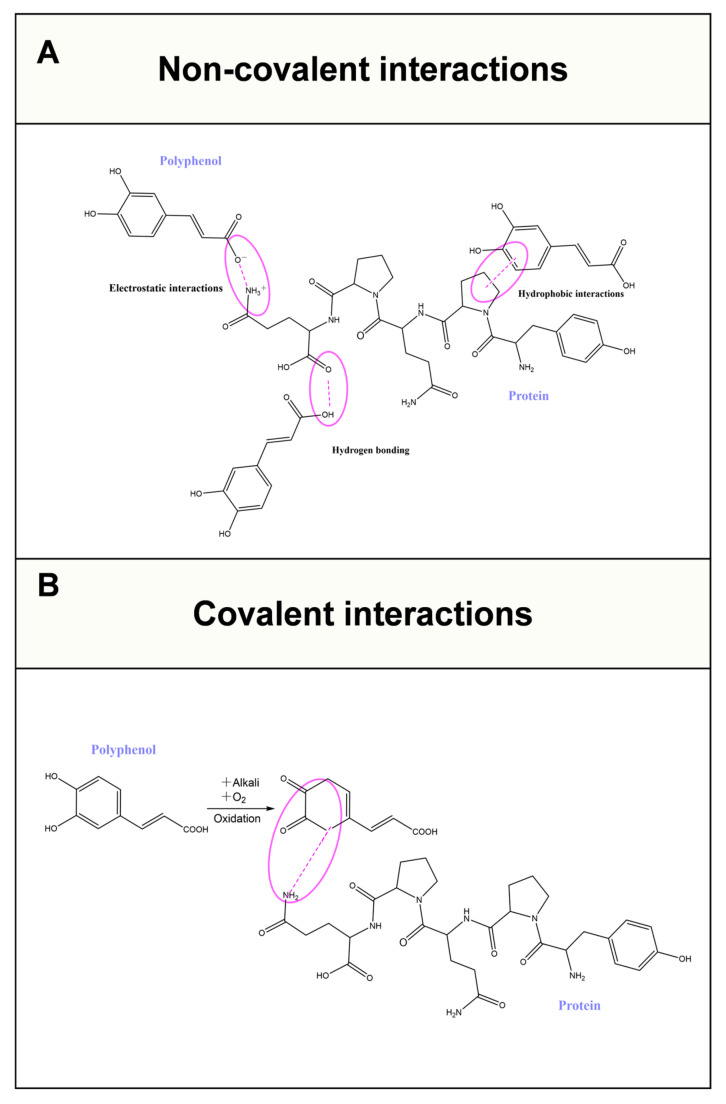
Schematic illustrations of the mechanisms of non-covalent/covalent interactions between water-soluble proteins and polyphenols. (**A**) The non-covalent interactions include hydrophobic interactions, hydrogen bonding, and electrostatic interactions. (**B**) The covalent interactions formed through oxidation reactions.

Polyphenols, as secondary metabolites produced by a wide variety of plants, have been proven to prevent many chronic diseases [42]. Due to the presence of aromatic rings and phenolic hydroxyl groups in their molecular structure, polyphenols can act as multidentate ligands to form complexes with water-soluble proteins. Generally, water-soluble proteins with compact, well-folded structures show limited binding affinity for polyphenols, whereas proline-rich proteins (e.g., gelatin) with disordered, random coil conformations exhibit much stronger complexation ability. Either non-covalent interactions (including hydrophobic interactions, hydrogen bonding, and electrostatic interactions) or covalent interactions (formed through oxidation reactions) can be involved in the formation of water-soluble protein–polyphenol complexes, depending on the complexing conditions [43,44]. Among the non-covalent interactions, hydrophobic interactions are the primary driving force in the formation of water-soluble protein–polyphenol complexes, and are enhanced by hydrogen bonding [45,46]. In contrast, electrostatic interactions play a relatively minor role, primarily because water-soluble proteins carry fewer charges at or near their isoelectric points, resulting in weak electrostatic interactions [47]. The mechanisms of non-covalent/covalent interactions between water-soluble proteins and polyphenols are summarized in Box 2 and illustrated in Figure 4.

Box 2 **The mechanisms of non-covalent/covalent interactions between water-soluble proteins and polyphenols.**

- Hydrophobic interactions occur between the hydrophobic residues of water-soluble proteins and the aromatic rings of polyphenols (Figure 4A) [45].
- Hydrogen bonding occurs between the carbonyl groups of water-soluble proteins and the hydroxyl groups of polyphenols (Figure 4A) [46].
- Electrostatic interactions occur between the positively charged amidogens of water-soluble proteins and negatively charged ester groups of polyphenols (Figure 4A) [47].
- Covalent interactions occur between the nucleophilic residues of water-soluble proteins and the semi-quinone radicals of polyphenols (Figure 4B) [15].

Controlled interactions between water-soluble proteins and polyphenols can be utilized to form complexes to enhance the stability and functionalities of water-soluble proteins. The incorporation of hydrophobic ester groups of polyphenols can enhance the hydrophobicity of water-soluble proteins, thereby enhancing their emulsifying properties [10]. Moreover, the complexation of water-soluble proteins with polyphenols is an exothermic process [48]. Therefore, it needs more energy to denature the water-soluble proteins in complexes, thus enhancing the thermal stability of water-soluble proteins. It has also been reported that polyphenols can act like proteins, with chaperone-like activity, to enhance the thermal stability of water-soluble proteins. Additionally, other studies have also shown that the complexation with polyphenols enhances the gelation [49] and foaming [50] properties of water-soluble proteins. Furthermore, the antioxidant activity of water-soluble proteins can also be enhanced by complexation with polyphenols [51]. However, unlike polysaccharides, complexation with polyphenols barely enhances the aggregation stability of water-soluble proteins at pHs near their isoelectric points. This is because both water-soluble protein and polyphenol carry little charge at pHs near the isoelectric points of water-soluble proteins, resulting in the incorporation of polyphenols that can hardly provide sufficient repulsive force to prevent the aggregation of water-soluble proteins near their isoelectric points. Therefore, incorporating polyphenols has little effect on water-soluble protein solubility at or near their isoelectric points. The main types of interactions and enhancements in other stability and functionalities of water-soluble proteins after complexation with polyphenols are summarized in Table 2.

### 2.4. Water-Soluble Protein–Small-Molecule Surfactant Complexes

Small-molecule surfactants, consisting of a hydrophilic head and a hydrophobic tail, have been used as ligands to form complexes with water-soluble proteins, thereby enhancing their stability and functionalities [63]. The reported water-soluble protein–small-molecule surfactant complexes are typically formed through non-covalent interactions rather than covalent interactions [64]. These non-covalent interactions generally occur at pHs away from the isoelectric points of the water-soluble proteins, as small-molecule surfactants are unable to prevent protein aggregation at pHs near the isoelectric points, which in turn hinders their interaction with water-soluble proteins. Electrostatic interactions, hydrogen bonding, and hydrophobic interactions play an important role in the non-covalent interactions between water-soluble proteins and small-molecule surfactants [65]. Owing to the considerable structural diversity and lack of uniform characteristic motifs in both the hydrophilic and hydrophobic moieties of small-molecule surfactants, it is still challenging to systematically elucidate the specific interaction groups involved in the complexation between water-soluble proteins and small-molecule surfactants.

The enhancement of the stability and functionalities of water-soluble proteins through complexation with small-molecule surfactants is predominantly manifested in thermal stability and emulsifying properties [66,67]. The enhancement of the emulsifying properties of water-soluble proteins is due to the fact that the complexation with a specific surfactant (such as lauryl arginine ethyl ester hydrochloride) induces water-soluble protein molecules to unfold, resulting in the exposure of more hydrophobic groups. The exposure of more hydrophobic groups leads to an enhancement of the hydrophobicity of water-soluble proteins, thus enhancing the emulsifying properties of water-soluble proteins. The enhancement of the thermal stability of water-soluble proteins is due to the fact that the secondary structural changes of water-soluble proteins during heating can be inhibited by complexation with specific surfactants (such as glycerin), thereby preventing protein aggregation during the thermal treatment induced by secondary structural changes [67]. Up to now, there are not many studies about water-soluble protein–surfactant complexes, which may be due to the concerns about the safety of small-molecule surfactants. For instance, rhamnolipid, as a common small-molecule surfactant with excellent surface activity, is often used to complex with water-soluble proteins to enhance their functionality. However, rhamnolipids have not yet been granted GRAS (generally recognized as safe) status, primarily because their main production strain, *Pseudomonas aeruginosa*, is an opportunistic pathogen that may pose a risk of endotoxin contamination. Recent studies have explored alternative production strains (e.g., *Pseudomonas putida*, *Burkholderia thailandensis*, or *genetically modified E. coli*) to improve safety profiles. Nevertheless, until these production methods are widely validated and recognized by regulatory authorities, the use of water-soluble protein–rhamnolipid complexes in food systems should be approached with caution, and comprehensive toxicological assessments are needed to support their safety.

## 3. Water-Soluble Protein-Based Ternary and Other Complexes

Although the stability and functionalities of water-soluble proteins can be enhanced through complexation with various ligands, the enhancement achieved by complexation with an individual ligand remains limited due to the intrinsic constraints associated with the nature of individual ligands. For instance, complexation with polysaccharides can enhance the aggregation stability of water-soluble proteins at pHs near their isoelectric points but has little effect on their antioxidant activity. Another example is that, while complexation with polyphenols or surfactants can significantly enhance the emulsifying properties of water-soluble proteins, it does not markedly enhance their aggregation stability at pHs near their isoelectric points. Therefore, multiple ligands have been used to complex with water-soluble proteins for simultaneously enhancing their stability and diverse functionalities [68].

**Figure 5:**
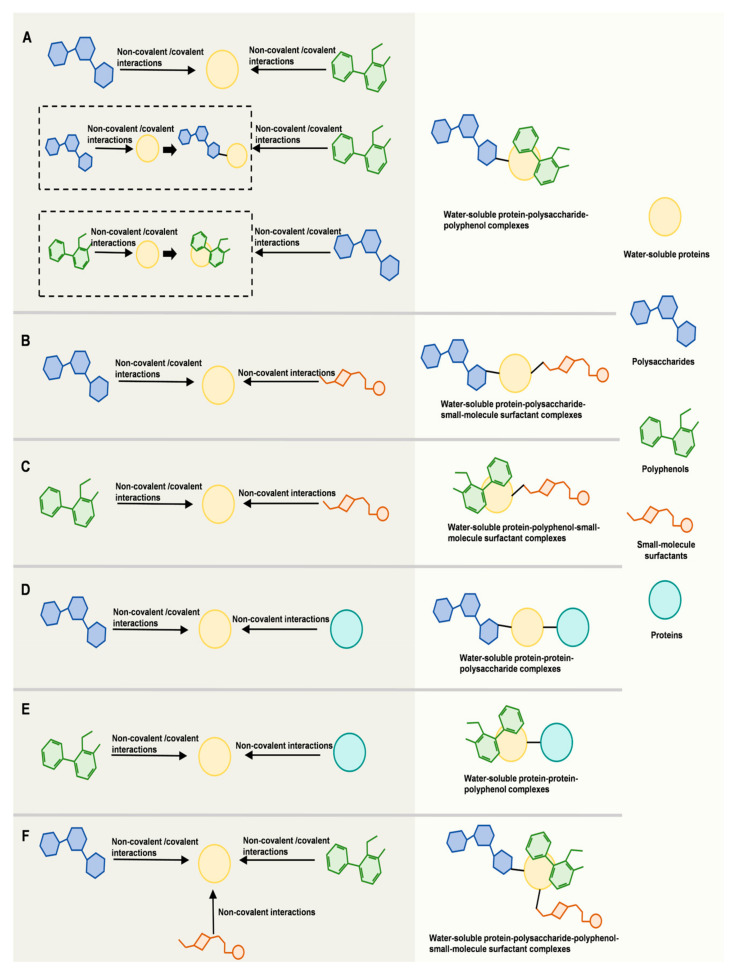
Conceptual illustration depicting both the interactions between water-soluble proteins and multiple ligands, and the subsequent formation of multielement complexes. (**A**) Water-soluble protein–polysaccharide–polyphenol ternary complexes formed through non-covalent/covalent interactions among the three components. (**B**) Water-soluble protein–polysaccharide–small-molecule surfactant ternary complexes formed through non-covalent/covalent interactions among the three components. (**C**) Water-soluble protein–polyphenol–small-molecule surfactant ternary complexes formed through non-covalent/covalent interactions among the three components. (**D**) Water-soluble protein–protein–polysaccharide ternary complexes formed through non-covalent/covalent interactions among the three components. (**E**) Water-soluble protein–protein–polyphenol ternary complexes formed through non-covalent/covalent interactions among the three components. (**F**) Water-soluble protein–polysaccharide–polyphenol–small-molecule surfactant quaternary complexes formed through non-covalent/covalent interactions among the four components.

Water-soluble protein-based multielement complexes have been successfully fabricated through either non-covalent or covalent interactions (Figure 5). The interactions induced the formation of water-soluble protein-based binary complexes also play an important role in the formation of water-soluble protein-based multielement complexes. Pre-binding of a ligand to the water-soluble protein may hinder or facilitate the complexation with subsequent ligands. Additionally, pre-binding may induce structural changes in the water-soluble proteins, which could alter the original interaction types with subsequent components. Due to the dynamic, multi-step, and interdependent nature of these interactions, elucidating the detailed mechanism of multielement complex formation remains a significant challenge. To address this challenge, a combination of advanced experimental and computational strategies is recommended. For instance, in addition to conventional techniques commonly employed to study the formation mechanisms of water-soluble protein-based complexes (such as isothermal titration calorimetry, circular dichroism, Fourier transform infrared spectroscopy, and others), the incorporation of time-resolved and in situ analytical methods enables the capture of transient intermediates and dynamic structural transitions during complexation. Simultaneously, molecular dynamics simulations provide atomistic and molecular-level insights into interaction mechanisms and conformational evolution, effectively complementing the spatial and temporal limitations of experimental approaches. On this basis, machine learning algorithms can be applied to extract underlying regularities and construct predictive models from large-scale, multidimensional experimental data, thus enabling a comprehensive elucidation of the complex formation mechanisms of water-soluble protein-based multicomponent systems.

At present, it has been demonstrated that the complexation of water-soluble proteins with multiple ligands can simultaneously enhance their stability and various functionalities. In our previous study, we found that using a polysaccharide (high methoxyl pectin) and a small-molecule surfactant (rhamnolipid) as ligands to simultaneously complex with a water-soluble protein (pea protein isolate) not only enhanced the aggregation stability of the protein but also enhanced its emulsifying properties. Based on this finding, we further incorporated a polyphenol (curcumin) into the complexes, resulting in an additional enhancement of the antioxidant activity of the protein [69].

## 4. The Factors Affecting the Enhancement of the Stability and Functionalities of Water-Soluble Proteins

The interactions of water-soluble proteins with ligands, as well as the enhancement of the stability and functionalities of water-soluble proteins, are mainly influenced by the nature of ligands and complexing conditions (including mass ratios of water-soluble proteins to ligands, pH, ionic strength, and temperature).

### 4.1. The Nature of Ligands

The nature of ligands influences both the type and strength of their interactions with water-soluble proteins. In our previous study, we selected rhamnolipid and lauryl arginine ethyl ester hydrochloride as representative anionic and cationic surfactants to form complexes with pea protein isolate and curcumin at pH 7, respectively. The results showed that hydrogen bonding and hydrophobic interactions were involved in both systems, while additional electrostatic interactions occurred only in the system containing lauryl arginine ethyl ester hydrochloride [6]. Another study demonstrated that the interactions between bovine serum albumin and sodium decyl sulfonate (an anionic surfactant) were stronger than those between bovine serum albumin and decyl triethylammonium bromide (a cationic surfactant) [70]. Moreover, it has been confirmed that the nature of ligands affects the degree of enhancement of the stability and functionality of water-soluble proteins through complexation. For instance, the enhancement of stability and emulsifying properties of water-soluble proteins depends on the chain length of the surfactants they form complexes with. Specifically, longer surfactant molecular chains lead to greater enhancement of both emulsifying properties and thermal stability.

### 4.2. Complexing Conditions

Generally, the type of interaction (non-covalent or covalent) is scarcely affected by the mass ratio of water-soluble proteins to ligands. However, it has been demonstrated to influence the enhancement of the stability and functionalities of water-soluble proteins. For instance, the aggregation stability of whey protein isolate at pHs near its isoelectric point becomes greater with a decrease in the mass ratio between whey protein isolate and soluble soybean polysaccharide from 6:1 to 2:1. This phenomenon can be attributed to the increased electrostatic repulsion caused by a higher proportion of negatively charged soluble soybean polysaccharide as the mass ratio is decreased [2]. In another instance, the antioxidant activity of walnut protein isolate increased as the mass ratio between walnut protein isolate and ellagic acid/epigallocatechin-3-gallate decreased from 50:1 to 20:1 [71].

pH is a major factor affecting the structure and net surface charge of water-soluble proteins, thereby influencing their interactions with ligands, as well as enhancement of their stability and functionalities [72]. The effect of pH on the interaction between bovine serum albumin and sodium dodecyl sulfate has been explored, and the results show that the interaction between bovine serum albumin and sodium dodecyl sulfate is promoted by the reduction of pH from 7.0 to 4.0. This is because, during the decrease in pH, the charge of bovine serum albumin gradually shifts from negative to positive, and its molecular structure gradually unfolds, exposing more hydrophobic groups, thereby promoting electrostatic and hydrophobic interactions with the sodium dodecyl sulfate [73]. An interesting result found is that the aggregation stability of lentil protein can be effectively enhanced by complexation with carboxymethyl cellulose in the pH range of 3.8–6.8, whereas in the pH range of 1.8–3.8, the aggregation stability of lentil protein cannot be effectively enhanced [25].

The interactions between water-soluble proteins and ligands are affected by the presence of ions in the systems. The presence of ions generally weakens the electrostatic interactions between water-soluble proteins and oppositely charged ligands through charge screening effects [74]. However, it can facilitate interactions between water-soluble proteins and ligands of the same charge through the bridging effect [75]. The extent to which ions affect the interactions between water-soluble proteins and ligands depends on their concentration. For instance, at low concentrations of sodium chloride (<10 mmol/L), the effect of it on water-soluble protein-based complexes is unobvious, but the effect becomes more pronounced as its level increases [76]. At a critical concentration of salt solutions, the presence of salts facilitates the interactions between water-soluble proteins and ligands by providing more available sites for electrostatic interactions and eliminating short-range repulsions between them [77].

**Figure 6:**
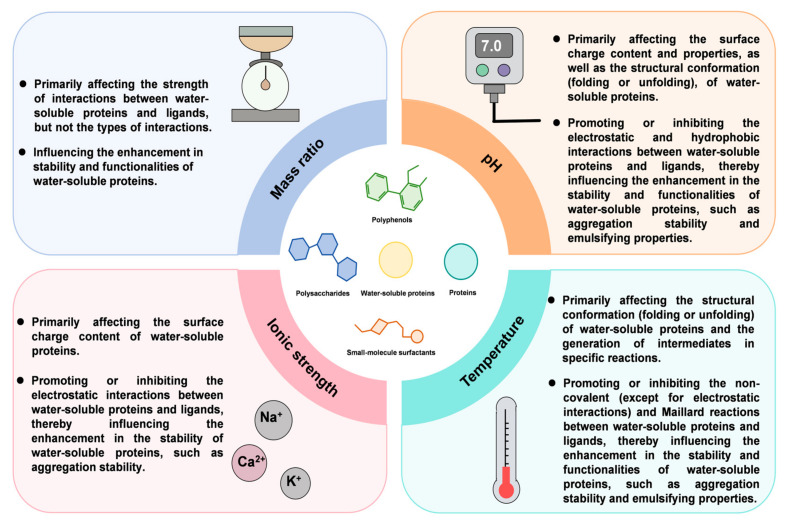
The influence of complexing conditions on the interactions between water-soluble proteins and ligands, as well as on the enhancement of their stability and functionalities.

Complexing temperature is another factor affecting the interactions between water-soluble proteins and ligands. Although the complexing temperature affects hydrophobic interactions, hydrogen bonding, and covalent bonding, it has little effect on electrostatic interactions [78]. This is because the net surface charge of water-soluble proteins and ligands does not change significantly with temperature. The hydrogen bonding between water-soluble proteins and ligands at low temperatures is stronger than that at high temperatures. This phenomenon is due to the fact that the motion of molecules increases in intensity with the increase of temperature, thus weakening hydrogen bonding and even leading to the breakage of hydrogen bonds. However, increasing temperature can lead to the conformational unfolding of water-soluble protein molecules, which may, on the one hand, promote hydrophobic interactions between water-soluble proteins and ligands [79], and on the other hand, it may also cause protein aggregation, thereby inhibiting their interactions with ligands. In addition, elevated temperature may enhance covalent interactions by promoting the Maillard reaction. The enhancement of interactions between water-soluble proteins and ligands induced by increased temperature may contribute to the enhancement of the stability and functionalities of water-soluble proteins; conversely, if the interactions are inhibited, it may hinder the enhancement of their stability and functionalities. The influence of complexing conditions on interactions between water-soluble proteins and ligands, as well as on the enhancement of the stability and functionalities of water-soluble proteins, is summarized in Figure 6 for easier comparison.

## 5. Applications of Water-Soluble Protein-Based Complexes

### 5.1. Stabilizers for Emulsions

There has been a growing interest in emulsion-based delivery systems to encapsulate non-polar bioactive components. As emulsion stabilizers, the emulsifiers should be carefully selected to ensure the physical and chemical stability of the emulsions. Up to now, various organic and inorganic emulsifiers have been developed to stabilize emulsions, such as polystyrene and silicon dioxide [80]. However, these emulsifiers are not desirable in the food industry due to concerns about toxicity, biocompatibility, and high cost. As a result, it is necessary to develop food-grade emulsifiers to stabilize the emulsions [80]. Of the alternative emulsifiers, many water-soluble protein-based complexes have been proved to stabilize the emulsions, such as soybean protein isolate–gellan gum complexes [81] and pea protein isolate–grape seed proanthocyanin complexes [62]. Compared to the binary complexes, the protein–polysaccharide–polyphenol or protein–polysaccharide–surfactant ternary complexes have been shown to be more suitable for stabilizing emulsions, since polyphenols and surfactants in complexes have been proposed to significantly reduce interfacial tension, resulting in the rapid and effective adsorption of complexes onto oil droplets [69,82]. Moreover, Feng et al. found that the lipid hydroperoxides concentration of the emulsion stabilized by pea protein isolate–high methoxyl pectin–epigallocatechin gallate complexes was 22.04 μmol/g oil after 15 days of storage at 45 °C, which was lower than that of the emulsion stabilized by pea protein isolate-high methoxyl pectin complexes (30.40 μmol/g oil) [83]. This result indicates that the emulsions stabilized by protein–polysaccharide–polyphenol complexes exhibit better oil oxidation stability.

### 5.2. Encapsulation

Water-soluble protein-based complexes are also used to encapsulate target ingredients that are sensitive to processing and storage conditions, allowing for the preservation of physicochemical and sensory properties. Studies have shown that these complexes effectively prevent the degradation of the target ingredients when exposed to environmental stresses [84]. Additionally, some complexes are stable in gastric acid, thereby protecting the target ingredient from low pH environments, leading to the slow release of the target ingredient [85]. In order to achieve more accurate targeted release, researchers are trying to graft the substances that have specific binding properties onto water-soluble protein-based complexes. The water-soluble protein-based complexes are also used to minimize the toxic effects associated with the use of metal-based carriers [86].

### 5.3. Potential Industrial Scalability and Commercial Examples

Currently, some water-soluble protein-based binary complexes have been successfully commercialized in products such as beverages and sauces, owing to their ease of preparation, good formulation stability, and regulatory compliance. For instance, the Purity Gum^®^ series developed by Ingredion, a soy protein isolate–polysaccharide complex, is widely used in dairy beverages, condiments, and baked goods to enhance emulsion stability and improve texture. Similarly, a whey protein isolate–pectin complex developed by Kerry Group is employed as a natural stabilizer in sports nutrition drink formulations. In contrast, despite their superior stability and functionality, water-soluble protein-based multielement complexes still face challenges in process control and scalability, having yet to reach commercialization. With the continuous growth of market demand, water-soluble protein-based multielement complexes are showing increasingly broad application prospects. In addition, improvements in process control are expected to further accelerate their industrial implementation.

## 6. Conclusions and Future Perspectives

Complexation between water-soluble proteins and ligands occurs through non-covalent or covalent interactions under controlled conditions. After complexation, the stability and functionalities of the water-soluble proteins are significantly enhanced, and the water-soluble protein-based complexes show a broad application prospect. However, the interaction mechanisms between water-soluble proteins and multiple ligands are still confusing. Moreover, the safety of water-soluble protein-based complexes remains a concern, not because of the components used to create the complexes but because of the deposition, distribution, and accumulation after delivery. Specifically, after depositing on the targeted organs, complexes may cause the generation of free radicals. The free radicals produced may pass through the cell membrane and react with cellular or subcellular biomacromolecules, causing cell damage. Furthermore, the effect of water-soluble protein-based complexes on the flavor and shelf life of foods must be considered. Last but not least, the nutritional effects of water-soluble protein-based complexes need to be further investigated through in vivo studies, particularly regarding their impact on gut microbiota and bioavailability, as disturbances in gut microbiota are generally associated with the occurrence of chronic diseases. Overall, further research is needed in this field to facilitate the broader application of water-soluble proteins in the food industry.

## Figures and Tables

**Table 1:** The interaction types involved in representative water-soluble protein–polysaccharide complexation processes and the enhancement of the stability and functionalities of water-soluble proteins.

Protein	Polysaccharide	Interaction Type	Enhancement of the Stability and Functionalities	References
Whey protein isolate	High methoxyl pectin	Electrostatic interaction and hydrophobic interaction	Thermal stability	[27]
Whey protein isolate	Tremella fuciformis polysaccharide	Electrostatic interaction	Digestive and storage stability	[28]
Whey protein isolate	Arabinoxylan	Covalent interaction	Emulsifying properties, thermal, pH, storage stability	[29]
Whey protein isolate	Dextran	Covalent interaction	pH, and ionic stability	[30]
β-lactoglobulin	Beet pectin	Electrostatic interaction	Aggregation stability	[31]
β-lactoglobulin	κ-carrageenan	Electrostatic complexation	Emulsifying and foaming properties	[32]
Soy protein	Potato starch	Electrostatic interaction	Emulsifying properties	[33]
Soy protein	Carrageenan	Electrostatic interaction	Digestive stability	[34]
Soy protein	Gum acacia	Covalent interaction	Aggregation stability and emulsifying properties	[35]
Pea protein	Tragacanth gum	Electrostatic interaction	Digestive stability	[36]
Pea protein	Soy soluble polysaccharide	Hydrogen bonding and hydrophobic interaction	Emulsifying and foaming properties	[37]
Pea protein	Inulin	Covalent interaction and hydrogen bonding	Thermal stability, emulsifying, and foaming properties	[38]

**Table 2:** The interaction types involved in representative water-soluble protein–polyphenol complexation processes and the enhancement of the stability and functionalities of water-soluble proteins.

Protein	Polyphenol	Interaction Type	Enhancement of the Stability and Functionalities	References
Whey protein isolate	Chlorogenic acid	Hydrophobic and electrostatic interaction	Foaming properties and antioxidant activity	[52]
Whey protein isolate	Gallic acid	Non-covalent interaction	Stability, foaming, and emulsifying properties	[53]
Whey protein isolate	Epigallocatechin gallate	Covalent interaction	Emulsifying properties and antioxidant activity	[54]
Whey protein isolate	Quercetin	Covalent interaction	Antioxidant activity, emulsifying properties, and thermal stability	[55]
β-lactoglobulin	Chlorogenic acid	Hydrogen bonding and hydrophobic interaction	Thermal stability and antioxidant activity	[56]
Egg white protein	Tea polyphenol	Electrostatic interaction	Emulsifying properties and antioxidant activity	[57]
Egg white protein	Caffeic acid	Covalent interaction	Emulsifying properties and antioxidant activity	[58]
Soy protein	Catechin	Hydrogen bonding and hydrophobic interaction	Emulsifying properties	[59]
Soy protein	Epigallocatechin gallate	Covalent interaction	Thermal stability and antioxidant activity	[60]
Pea protein	Epigallocatechin gallate	Hydrogen bonding and van der Waals forces	Emulsifying properties and foaming properties	[61]
Pea protein	Grape seed proanthocyanidin	Hydrogen bonding and hydrophobic interaction	Storage stability	[62]

## Data Availability

No new data were created.
